# Functionally significant polymorphisms of the *MMP-9* gene are associated with peptic ulcer disease in the Caucasian population of Central Russia

**DOI:** 10.1038/s41598-021-92527-y

**Published:** 2021-06-29

**Authors:** Oksana Minyaylo, Irina Ponomarenko, Evgeny Reshetnikov, Volodymyr Dvornyk, Mikhail Churnosov

**Affiliations:** 1 Department of Medical Biological Disciplines, Belgorod State University, 308015 Belgorod, Russia; 2 Department of Life Sciences, College of Science and General Studies, Alfaisal University, Riyadh, 11533 Saudi Arabia

**Keywords:** Genetics, Genetic association study

## Abstract

This study analyzed the association of functionally significant SNPs of matrix metalloproteinase (*MMP*) genes in the development of peptic ulcer disease (PUD) in Caucasians from Central Russia. Ten SNPs of the *MMP-1, MMP-2, MMP-3, MMP-8*, and *MMP-9* genes were analyzed for association with PUD in a cohort of 798 patients with PUD (including 404 *H. pylori*-positive and 394 *H. pylori*-negative) and 347 *H. pylori*-negative controls using logistic regression and assuming the additive, recessive, and dominant genetic models. The variants of *MMP-1*, *MMP-2*, *MMP-3*, and *MMP-8* did not manifest any significant associations with the diseases. Five SNPs of the *MMP-9* gene demonstrated such association. Allele G of the rs17576 *MMP-9* locus conferred a higher risk for PUD (OR_adj_ = 1.31, p_perm_ = 0.016), haplotype AACG of loci rs17576-rs3787268-rs2250889-rs17577 of the *MMP-9* gene decreased risk for PUD (OR_adj_ = 0.17, p_perm_ = 0.003). Also, allele C of rs3918249, allele G of rs17576 and haplotype CG of rs3918249-rs17576 of the *MMP-9* gene increased risk for *H. pylori*-positive PUD (OR_adj_ = 1.82, p_perm_ = 0.002; OR_adj_ = 1.53–1.95 p_perm_ = 0.001–0.013 and OR_adj_ = 1.49 p_perm_ = 0.009 respectively). The above loci and 50 linked to them possess significant regulatory effects and may affect the alternative splicing of four genes and the expression of 17 genes in various organs and tissues related to the PUD pathogenesis.

## Introduction

Peptic ulcer is the cyclical appearance of a limited mucosal defect in the digestive tract (usually the stomach or duodenum) extending deeply beyond the muscular plate of the mucous membrane, with inflammatory infiltration and thrombotic necrosis in adjacent tissues^1^. The prevalence of peptic ulcer disease (PUD) in the general population is estimated at 5–10%^2^.

Mucosal defects in patients with the acid peptic disease have been traditionally considered as a result of increased gastric acid secretion in the stomach and degradation of the mucus barrier^2–4^. Risk factors for PUD, including gastric and duodenal ulcers, are infection by *H. pylori*, alcohol and tobacco consumption, use of non-steroidal anti-inflammatory drugs (NSAIDs) and aspirin, stress, etc.^2–6^. However, only a relatively small proportion of people infected by *H. pylori* or using NSAIDs develop PUD that suggests variation in individual susceptibility to the beginning of mucosal damage^7^. On the other hand, about one-fifth of cases include *H. pylori*-negative, NSAID-negative, and aspirin-negative PUD collectively classified as an idiopathic ulcer^8^. This type of ulcer is thought to occur due to the imbalance between factors important for mucosal integrity and aggressive insults, but the exact pathogenic mechanisms of idiopathic peptic ulcer remain unknown^3^.

Matrix metalloproteinases (MMPs) are endopeptidases playing an important role in the extracellular matrix (ECM) remodeling, cell proliferation, and inflammation. MMPs are synthesized and secreted by gastric and duodenal epithelial cells, macrophages, and neutrophils^9^. Since ECM degradation is an important factor of gastric and duodenal mucosal damage and subsequent PUD, MMPs play a key role in this process^9–11^. There is evidence that cleaving and remodeling of the ECM by MMPs is one of the factors contributing to gastric ulceration (GU)^12,13^. The role of several MMPs (MMP-1, MMP-2, MMP-3, MMP-9, and MMP-13) in GU was studied using animal models^13–16^. MMP-9 was shown to be important in the early phase of chronic GU^16^.

Several genes have been reported for their association with peptic ulcers^7,17–19^. Polymorphisms of the *MMPs* genes (*MMP-9, MMP-7, MMP-3*) may contribute to a genetic risk profile for gastric and duodenal ulcers in chronic *H. pylori* infection^17–19^. *H. pylori* infection can induce the expression of MMP-3, MMP-7, and MMP-9 in the gastric mucosa and sera^18,20,21^. *MMP-9* was significantly up-regulated in *H. pylori*-positive as compared to *H. pylori*-negative GU^22^.

Despite the apparently significant role of MMPs in PUD pathogenesis, associations of MMP genetic variants with PUD have been poorly analyzed: only a few studies of this problem have been published so far^17–19,23^. Shaimardanova et al.^17^ reported associations of polymorphic variants of the *MMP-1* (rs1799750, rs494379), *MMP-3* (rs3025058), *MMP-9* (rs17576) genes with PUD in Tatars of Russia but no such association was found for rs3918242 of the *MMP-9* gene. Hellmig et al.^19^ documented rs11568818 of *MMP-7* and rs17576 of *MMP-9* as risk factors of *H. pylori*-positive GU in Germans. On the contrary, Yeh et al.^18^ did not find the association between rs17576 and *H. pylori*-positive gastric/duodenal ulcer in Taiwanese females. Likewise, no statistically significant associations of rs3918242 *MMP-9* with the duodenal ulcer in children in the Chinese population were found^23^. Overall. this prompts for filling in this gap.

The present study analyzed polymorphisms of *MMP-1, MMP-2, MMP-3, MMP-8*, and *MMP-9* genes for their association with PUD and possible role in the susceptibility to the disease in the Caucasian sample from the Central Region of Russia.

## Results

The phenotypic data of the study participants are shown in Table 1. The PUD patients had a more common family history of peptic ulcer (p = 0.0005), alcohol (p = 0.0005) and tobacco (p = 0.0005) consumption, stress (p = 0.0005), the presence of cardiovascular pathology (p = 0.0005) *versus* the control group. These parameters were used as confounding factors (covariates) in the regression association analyses.

**Table 1:** Phenotypic characteristics of the study participants.

Parameters	Control mean ± SD, % (n)	PUD mean ± SD, % (n)	p
N	347	798	–
Age, years (min–max)	48.47 ± 13.69 (22–79)	48.54 ± 14.28 (20–79)	0.92
Gender ratio, f/m	66.28/33.72 (230/117)	67.42/32.58 (538/260)	0.76
BMI, kg/m^2^	26.83 ± 5.09	26.94 ± 5.30	0.78
Age of developing peptic ulcer, years	–	41.12 ± 12.87	–
Family history of peptic ulcer	4.32 (15)	18.29 (146)	**0.0005**
Current smoking	14.99 (52)	33.08 (264)	**0.0005**
Alcohol consumption	32.28 (112)	51.13 (408)	**0.0005**
Stress	37.17 (129)	77.19 (616)	**0.0005**
Positivity *H. pylori* test (endoscopic biopsy and histological identification)	–	50.63 (404)	–
**PUD characteristics**			
Location			
Stomach: body	–	2.76 (22)	–
Pylorus	–	3.01 (24)	–
Antrum	–	48.62 (388)	–
Duodenum: bulb	–	45.61 (364)	–
Sizes ulcer (diameter) (cm)	–	0.61 ± 0.40	–
Sizes ulcer: small (< 0.5 cm)	–	45.37 (362)	–
Medium (0.5–1.0 cm)	–	44.86 (358)	–
Large (> 1.0 cm)	–	9.77 (78)	–
**PUD associated complications**			
Bleeding	–	3.51 (28)	–
Perforation	–	8.27 (66)	–
Stenosis	–	6.52 (52)	–
Malignancy	–	2.26 (18)	–
**Other somatic pathologies**			
Cardiovascular pathology	26.80 (93)	48.37 (386)	**0.0005**
Endocrine pathology	3.17 (11)	5.01 (40)	0.22
Kidney pathology	2.59 (9)	3.76 (30)	0.41
Respiratory system pathology	4.32 (15)	5.76 (46)	0.39
Nervous system pathology	7.78 (27)	9.52 (76)	0.40
Musculoskeletal system pathology	6.91 (24)	8.02 (64)	0.60

p values < 0.05 are shown in bold.

Supplementary Table S1 shows distributions of genotypes and alleles of the ten studied SNPs in the PUD patients and control groups. All analyzed SNPs were in the HWE (p > 0.005, p_bonf_ > 0.05). The analysis yielded no significant associations for all the studied SNPs but one of the *MMP-9* gene with PUD (Table 2). Specifically, the increased risk of PUD was associated with allele G of SNP rs17576 *MMP-9* (additive model, the odds ratio adjusted for confounding factors OR_adj_ = 1.31, p_perm_ = 0.016, power—82.98%) (Table 2). Besides, two loci of the *MMP-9* gene (rs3918249 and rs17576) were individually associated with *H. pylori*-positive PUD (Table 3). Allele C of SNP rs3918249 showed a significant association with the increased risk of *H. pylori*-positive PUD (dominant model, OR_adj_ = 1.82, p_perm_ = 0.002, power—96.43%). The increased risk of *H. pylori*-positive PUD was also associated with a carriage of allele G of loci rs17576 according to the all three genetic models: additive (OR_adj_ = 1.53, p_perm_ = 0.001, power—98.14%), dominant (OR_adj_ = 1.67, p_perm_ = 0.013, power—90.21%), recessive (OR_adj_ = 1.95, p_perm_ = 0.007, power—94.75%).

**Table 2:** Associations of the *MMP* gene polymorphisms with PUD.

SNP	Gene	MAF	n	Additive model				Dominant model				Recessive model			
				OR	95% CI		P	OR	95% CI		P	OR	95% CI		P
					L95	U95			L95	U95			L95	U95	
rs1940475	*MMP-8*	T	1136	0.96	0.79	1.18	0.708	0.91	0.66	1.26	0.573	0.99	0.71	1.39	0.960
rs1799750	*MMP-1*	2G	1107	0.89	0.73	1.09	0.263	0.86	0.62	1.19	0.362	0.84	0.59	1.02	0.345
rs679620	*MMP-3*	T	1133	0.97	0.79	1.20	0.797	0.93	0.66	1.30	0.655	1.01	0.72	1.41	0.979
rs243865	*MMP-2*	T	1121	0.96	0.76	1.22	0.749	0.94	0.69	1.27	0.672	1.01	0.57	1.80	0.969
rs3918242	*MMP-9*	T	1127	1.00	0.75	1.32	0.973	1.06	0.77	1.46	0.733	0.58	0.22	1.52	0.266
rs3918249	*MMP-9*	C	1125	1.16	0.93	1.43	0.181	1.45	1.07	1.97	0.018	0.88	0.59	1.33	0.549
rs17576	*MMP-9*	G	1140	**1.31**	**1.05**	**1.60**	**0.016**	1.35	0.99	1.83	0.054	1.51	1.00	2.27	0.048
rs3787268	*MMP-9*	A	1133	1.12	0.87	1.45	0.384	1.17	0.86	1.58	0.315	1.02	0.48	2.14	0.968
rs2250889	*MMP-9*	G	1128	0.79	0.57	1.09	0.148	0.77	0.53	1.12	0.172	0.63	0.22	1.80	0.388
rs17577	*MMP-9*	A	1112	1.00	0.75	1.32	0.988	1.01	0.80	1.52	0.563	0.46	0.18	1.17	0.102

All results were obtained after adjustment for covariates.

p values < 0.017 are shown in bold.

*OR* odds ratio, *95% CI* 95% confidence interval.

**Table 3:** Associations of the *MMP* gene polymorphisms with *H. pylori*-positive and *H. pylori*-negative PUD.

SNP	Gene	MAF	n	Additive model				Dominant model				Recessive model			
				OR	95% CI		P	OR	95% CI		P	OR	95% CI		P
					L95	U95			L95	U95			L95	U95	
***H. pylori*-positive PUD**															
rs1940475	*MMP-8*	T	744	0.97	0.76	1.23	0.774	0.91	0.62	1.36	0.656	0.99	0.66	1.49	0.979
rs1799750	*MMP-1*	2G	725	0.88	0.69	1.13	0.313	0.83	0.56	1.23	0.361	0.85	0.55	1.31	0.452
rs679620	*MMP-3*	T	743	0.92	0.71	1.18	0.505	0.85	0.57	1.28	0.447	0.93	0.62	1.42	0.744
rs243865	*MMP-2*	T	735	0.98	0.74	1.30	0.879	0.90	0.63	1.30	0.588	1.26	0.64	2.46	0.509
rs3918242	*MMP-9*	T	739	1.17	0.83	1.63	0.376	1.34	0.92	1.96	0.127	0.30	0.06	1.39	0.123
rs3918249	*MMP-9*	C	737	1.33	1.03	1.72	0.031	**1.82**	**1.23**	**2.67**	**0.002**	1.03	0.63	1.67	0.914
rs17576	*MMP-9*	G	746	**1.53**	**1.19**	**1.98**	**0.001**	**1.67**	**1.14**	**2.43**	**0.008**	**1.95**	**1.22**	**3.11**	**0.005**
rs3787268	*MMP-9*	A	745	1.23	0.91	1.67	0.181	1.26	0.87	1.81	0.219	1.43	0.62	3.30	0.396
rs2250889	*MMP-9*	G	736	0.77	0.51	1.15	0.203	0.78	0.49	1.23	0.282	0.42	0.09	2.01	0.280
rs17577	*MMP-9*	A	728	1.20	0.86	1.68	0.271	1.43	0.98	2.09	0.067	0.37	0.10	1.35	0.132
***H. pylori*-negative PUD**															
rs1940475	*MMP-8*	T	738	0.98	0.77	1.25	0.893	0.94	0.63	1.39	0.752	1.02	0.68	1.53	0.920
rs1799750	*MMP-1*	2G	721	0.91	0.71	1.17	0.459	0.90	0.61	1.33	0.596	0.86	0.56	1.32	0.483
rs679620	*MMP-3*	T	735	0.86	0.67	1.10	0.235	0.79	0.53	1.19	0.258	0.84	0.55	1.28	0.419
rs243865	*MMP-2*	T	729	0.94	0.70	1.26	0.696	0.97	0.67	1.39	0.849	0.80	0.39	1.67	0.558
rs3918242	*MMP-9*	T	731	0.83	0.59	1.18	0.296	0.80	0.53	1.20	0.276	0.83	0.28	2.46	0.739
rs3918249	*MMP-9*	C	733	1.00	0.78	1.30	0.975	1.15	0.80	1.66	0.448	0.77	0.46	1.30	0.323
rs17576	*MMP-9*	G	740	1.08	0.83	1.40	0.569	1.09	0.76	1.57	0.626	1.12	0.67	1.88	0.660
rs3787268	*MMP-9*	A	733	1.01	0.74	1.40	0.930	1.09	0.75	1.57	0.656	0.62	0.21	1.77	0.367
rs2250889	*MMP-9*	G	734	0.81	0.55	1.20	0.293	0.78	0.49	1.22	0.268	0.82	0.25	2.07	0.739
rs17577	*MMP-9*	A	724	0.80	0.57	1.14	0.217	0.81	0.54	1.22	0.310	0.53	0.17	1.65	0.270

All results were obtained after adjustment for covariates.

p values < 0.017 are shown in bold.

*OR* odds ratio, *95% CI* 95% confidence interval.

**Figure 1:**
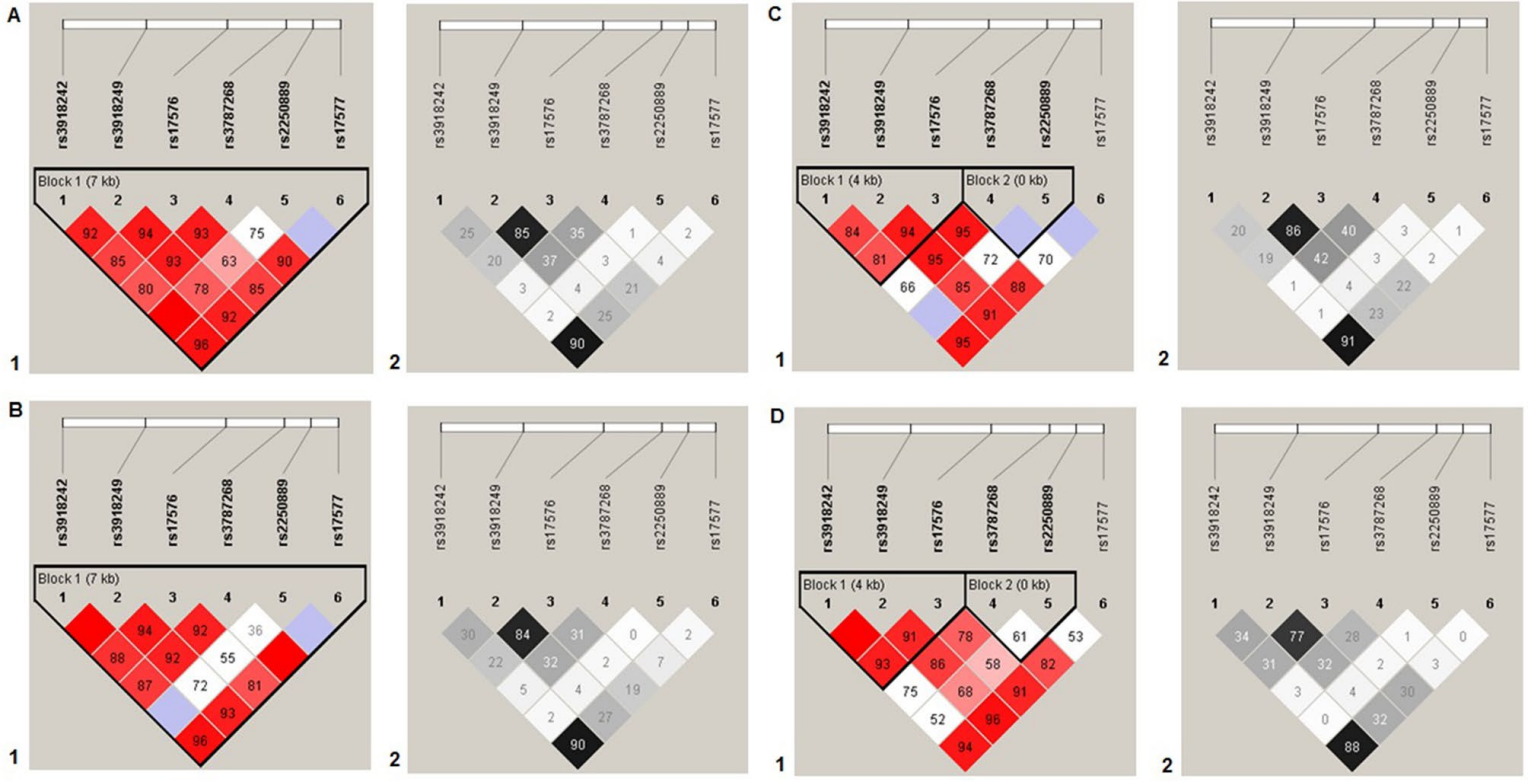
Linkage disequilibrium (LD) between SNPs rs3918242, rs3918249, rs17576, rs3787268, rs2250889, and rs17577 of the *MMP-9* gene. (**A**) All PUD patients, (**B**) *H. pylori*-positive PUD patients, (**C**) *H. pylori*-negative PUD patients, (**D**) control group. LD values are presented as Lewontin's standardized coefficient D′ (Figure Sects. 1) and the square of the correlation Pearson's coefficient (r^2^) (Figure Sects. 2) between the SNPs.

Haplotype AACG defined by rs17576-rs3787268-rs2250889-rs17577 was associated with PUD (OR_adj_ = 0.17, p = 0.001, p_perm_ = 0.003), haplotype CG defined by rs3918249-rs17576 of the *MMP-9* gene was associated with *H. pylori*-positive PUD (OR_adj_ = 1.49, p = 0.004, p_perm_ = 0.009) (Fig. 1). Thus, in total five polymorphisms of the *MMP-9* gene were associated with PUD (two individually and three within haplotypes).

### Functional SNP

#### Non-synonymous SNPs

Among the PUD-associated SNPs, three polymorphisms were missense: rs17576 (Gln279Arg), rs2250889 (Arg574Pro), and rs17577 (Arg668Gln) (Supplementary Table S2). According to the SIFT online tool, these loci have prediction values «tolerated» (rs17576 and rs2250889) and «deleterious» (rs17577) (Supplementary Table S2).

#### Regulatory effects

The data on the regulatory effects of the PUD-associated loci of the *MMP-9* gene are presented in Supplementary Table S3. According to the HaploReg database, three SNPs (rs17576, rs2250889, and rs17577) are located in evolutionarily conserved regions, all five polymorphisms—in the region of DNA binding with modified histone (H3K4me3, H3K9ac) marking promoters and hypersensitivity region to DNAse-1 in various tissues, four SNPs (rs17576, rs3918249, rs3787268, and rs2250889)—in the region of DNA binding with modified histone (H3K4me1, H3K27ac) marking enhancers and two polymorphisms (rs17577 and rs2250889)—in the protein-bound region. Importantly, the PUD-associated SNPs manifest their regulatory effects in the tissues and organs related to the pathogenesis of the disease (fetal stomach and small intestine, adult gastric and small intestine, adult stomach, and duodenum mucosa, etc.).

In addition to the five PUD-associated SNPs, regulatory significance was estimated for 50 polymorphisms linked to them (Supplementary Table S3). Three synonymous SNPs were located in exons of the *MMP-9* gene, 28 SNPs were in 5'-UTR of the *MMP9, ZNF335*, and *SLC12A5* genes, 19 were in introns. Ten loci were located in evolutionarily conserved regions. The in silico analysis of the linked SNPs suggested several polymorphisms with pronounced regulatory effects (Supplementary Table S3). For example, rs3848722, rs3848721, and rs13969 (were in linkage disequilibrium with SNPs rs3918249 and rs17576) are located in the hypersensitive region to DNAase-I (19, 20, and 24 tissues, respectively), in the region of DNA binding with modified histone marking promoters and enhancers (5 and 14 tissues respectively for rs3848722; 4 and 12 tissues respectively for rs3848721; 12 and 14 tissues respectively for rs13969), and a putative transcription factor binding sites (Pax-6, HNF4, ZID, NRSF for rs3848722; SP1, Zfp281, STAT for rs3848721; ATF3, E2F, XBP-1, p300 for rs13969). Also, the SNP rs13969 is situated in the protein-bound region (with this DNA region interact seven regulatory proteins—SMC3, CCNT2, HAE2F1, RAD21, ZEB1, CTCF, ZNF263) (Supplementary Table S3).

#### Expression QTLs

In silico analysis for the eQTL impact of the PUD-associated SNPs shows their might affect the expression of 17 genes (*MMP9, CD40, NTTIP1, NEURL2, PCIF1, PLTP, RP11-465L10.10, RP3-337O18.9, RPL13P2, SLC12A5, SNX21, SPATA25, SYS1, WFDC10B, WFDC3, ZNF335, ZSWIM1*) in more than 20 tissues and organs (Supplementary Table S4). For example, rs3918249 and rs17576 correlate with the transcription levels of various genes in the digestive organs (esophagus, colon) and other tissues and organs related to the pathophysiology of PUD: thyroid (*NEURL2*), adrenal gland (*PCIF1, SLC12A5, RP11-465L10.10)*, whole blood (*ZNF335*), adipose tissue (visceral and subcutaneous) (*SPATA25, NEURL2, PLTP, CD40, RP3-337O18.9, ZSWIM1)*, etc. (Supplementary Table S4) The PUD risk alleles G rs17576 and C rs3918249 determined in the present study downregulate the affected genes in most eQTL (Supplementary Table S4). The PUD-associated loci were also in strong LD with the 48 SNPs affecting the expression of the above 17 genes in various organs and tissues (Supplementary Table S5).

#### Splicing QTLs

The PUD-associated SNPs possessed sQTL with the potential impact on alternative splicing and might affect four genes (*PLTP, ACOT8, SNX21, SLC12A5*) (Supplementary Table S6). These loci were tightly linked to 48 polymorphisms affecting sQTL of the above four genes in more than 20 tissues and organs (Supplementary Table S7). Noteworthy is the data that independently associated with PUD and/or *H. pylori*-positive PUD SNPs, rs3918249 and rs17576, correlate with genes alternative splicing in various parts of the brain (cortex and substantia nigra of brain, pituitary, etc.) implicated in the pathophysiology of the disease. According to the results of the present study, allelic variants rs17576 and rs3918249) (alleles G and C respectively) may have a multidirectional effect in different parts of the brain (Supplementary Table S6). For example, allele C rs3918249 is associated with a low level of alternative splicing of the *SLC12A5* gene in the brain cortex (effect size β = − 0.44, p = 2.9e−7) and a high level of sQTL of the same gene in substantia nigra of brain (β = 0.61, p = 5.6e−7) and pituitary (β = 0.63, p = 3.0e−12). Similarly, allele G rs17576 correlates with a low sQTL value of the *SLC12A5* gene in brain cortex (β = −0.45, p = 3.3e−7) and a high sQTL value of this gene in the pituitary (β = 0.63, p = 9.3e−12) (Supplementary Table S6).

## Discussion

The present study reports for the first time the association of *MMP-9* gene polymorphisms with PUD in Caucasians from Central Russia: allele G of SNPs rs17576 locus increased risk for PUD (OR_adj_ = 1.31) whereas haplotype AACG of rs17576-rs3787268-rs2250889-rs17577 decreased the risk (OR_adj_ = 0.17). Also, allele C of rs3918249, allele G of the rs17576 and haplotype CG of rs3918249-rs17576 increased risk for the *H. pylori*-positive PUD (OR_adj_ = 1.82, OR_adj_ = 1.53–1.95 and OR_adj_ = 1.49 respectively). The PUD-associated loci appeared to possess significant regulatory effects and influence the expression of 17 genes and alternative splicing of four genes.

One of the PUD-associated loci, rs17576, was previously shown as a candidate for *H. pylori*-positive gastric ulcer^19^, peptic ulcer, and *H. pylori*-positive peptic ulcer^17^. However, the data about the risk alleles of this locus were contradictory, Specifically, Shaimardanova et al.^17^ reported allele G (i.e., the same as determined in the present study) as the risk factor for PUD and *H. pylori*-positive PUD in Tatars from the Bashkortostan Republic of Russia, whereas Hellmig et al.^19^ determined allele A as the risk factor for *H. pylori*-positive gastric ulcer in Germans. On the other hand, Yeh et al.^18^ did not find any association of rs17576 with either gastric or duodenal ulcer after *H. pylori* infection in Taiwanese. Okada et al.^24^ ported the association of rs17576 *MMP-9* c gastric cancer both individually and within haplotype CAA rs3918242-rs17576-rs17577 of the *MMP-9* gene.

The MMP-9 protein (gelatinase B) cleaves denatured collagen and plays a significant role in ECM modification^25^. MMPs can be induced by both *H. pylori* bacterial products and proinflammatory cytokines^26^. Overexpression of MMPs may result in extracellular matrix breakdown and tissue disintegration. Li et al.^22^ reported higher MMP-9 expression in the gastric mucosa at the boundary of the gastric ulcer. Significantly elevated expression of pro-MMP9 (about 12-fold) was documented in the indomethacin-induced gastric ulcer as compared to unaffected tissues. Ethanol produced an even stronger effect and increased pro-MMP-9 expression in rat gastric tissues up to 22-fold^14^. Overexpression of MMP-9 in indomethacin-induced gastric ulcer in mice correlated with up-regulation of activator protein-1 and preceded oxidative stress^27^.

During PUD, the gastric and duodenal mucosa is infiltrated by monocytes, lymphocytes, neutrophils, and plasma cells. Inflammatory cells produce multiple pro-inflammatory cytokines and growth factors (e.g., epidermal growth factor, transforming growth factor-β, platelet-derived growth factor, vascular endothelial growth factor, etc.)^2,22^. Pro-inflammatory cytokines can elevate the expression of MMPs^26^. Chronic inflammation precedes oxidative stress and increases the expression of MMP-9^22^.

We determined associations of the *MMP-9* gene polymorphisms with *H. pylori*-positive PUD but did not find the association of any of the analyzed *MMP* genes with *H. pylori*-negative PUD. The polymorphisms of the *MMP-9* gene may contribute to a complex genetic risk profile of PUD in chronic *H. pylori* infection^17,19^. Our results are in agreement with the previous reports about more significant contribution of *MMP-9* to the development of *H. pylori*-positive gastric ulcer and gastritis as compared to the other *MMP* genes^22,28–30^. Li et al.^22^ showed that *MMP-9* expression levels in the gastric mucosa were significantly elevated in *H. pylori*-positive gastric ulcer patients as compared to the *H. pylori*-negative ones and correlated with the histologically determined activity level and inflammation at the boundary of the ulcer. Epithelium of the *H. pylori*-induced gastric ulcer manifested higher *MMP-9* expression than that of the NSAID-related gastric ulcer^28^. Significantly higher serum levels of MMP-9 were determined in patients with *H. pylori*-positive gastritis as compared to *H. pylori*-negative controls^29^. Antral mucosa of *H. pylori*-infected patients with gastritis demonstrated a 19-fold higher MMP-9 protein activity and tenfold increase of the *MMP-9* gene expression than that in uninfected individuals^30^. Successful treatment of the *H. pylori* infection lowered the *MMP-9* expression levels, whereas the elevated levels remain unchanged when the treatment failed^31^.

It should be noted that the current study is somewhat limited because only one ethnic population was analyzed. The well-known ethnic disparities in the prevalence of complex diseases warrant validation studies of the determined associations of the *MMP* genes and PUD in other ethnic populations.

## Conclusions

Genetic variants of the gene are associated with PUD in a population of Central Russia. However, the data about the possible role of the *MMP* genes polymorphic variants in the susceptibility to PUD in different ethnic populations remain inconsistent that warrants further studies to identify possible causative variants for the disease.

## Methods

### Study subjects

In total, 1145 participants, including 798 patients with PUD (434 with gastric ulcer and 364 with the duodenal ulcer), and 347 controls, were recruited for the study. The inclusion criteria were as follows: Russian ethnicity (self-reported) and birthplace in Central Russia^32,33^, age of 20 and above, voluntary consent to participate in the study, a positive diagnosis of PUD (case group) or absence of the gastrointestinal disease (control group)^34^. PUD and complications (if any) were determined on the basis of conventional clinical and endoscopic findings. They were not examined by endoscopy because, apart from ethical reasons, the chance of finding an active ulcer in patients without symptoms was very low^35^. Individuals with chronic diseases of the vital organs (cardiovascular, respiratory, or kidney insufficiency), severe autoimmune disorders, and taking NSAIDs, corticosteroids, and aspirin for a long-term treatment were excluded from the study^34^.

The *H. pylori* infection in patients was diagnosed histologically (Giemsa stain^36^) in biopsies taken from the antrum and corpus of the stomach by the endoscopic procedure^35^. Among 798 patients with PUD, 404 were *H. pylori*-positive and 394 were *H. pylori*-negative. In the controls, the presence of *H. pylori* was diagnosed by the serological test using a commercial IgG ELISA kit (Plate Helicobacter IgG, Roche). Control group volunteers diagnosed with *H. pylori* infection were excluded from the study.

The study protocol was approved by the Medical Institution Ethics Committee of Belgorod State University. All participants signed an informed consent prior to enrolment in the study. All methods were performed following the relevant guidelines and regulations. The participants took the medical examination at the Department of Gastroenterology of St. Joasaph Belgorod Regional Clinical Hospital.

### Isolation of DNA and genotyping

A blood sample (4–5 ml) was collected by venipuncture from all study participants in EDTA-coated tubes (Vacutainer®). Genomic DNA was isolated from the buffy coat using a standard phenol/chloroform procedure (as described earlier^37^).

Ten SNPs of the *MMP* genes (rs1799750 *MMP-1,* rs243865 *MMP-2,* rs679620 *MMP-3,* rs1940475 *MMP-8,* rs3918242, rs3918249, rs3787268, rs2250889, rs17576, and rs17577 *MMP-9*) were selected for the analysis according to the following criteria^38,39^: previously reported associations with digestive diseases (PUD, gastric cancer, etc.), regulatory potential, and MAF > 0.05.

All selected SNPs had significant regulatory potential as evidenced by the HaploReg online tools^40^ (Supplementary Table S8); eight polymorphisms were associated with digestive diseases (PUD, gastric and esophageal cancer, digestive cancers, gastritis) (including two SNPs associated with PUD) in previously published candidate gene association studies (Supplementary Table S9). Two SNPs (rs3918249 and rs3787268 *MMP-9*) did not demonstrate a significant association with digestive diseases but had significant regulatory potential (according to HaploReg).

The polymorphisms were genotyped using the MALDI‐TOF mass spectrometry iPLEX platform (Agena Bioscience Inc, San Diego, CA). The quality was controlled by genotyping of blind replicates^41^. Regenotyping of 5% of the studied samples, selected on a random basis, showed 100% reproducibility of the original results.

### Statistical analysis

The observed allele and genotype frequencies were assessed for correspondence to the Hardy–Weinberg equilibrium using the chi-square test^42^. Associations of the SNPs with PUD were analyzed by logistic regression according to three main genetic models, additive, recessive, and dominant^43^. The regression analysis was adjusted for covariates: family history of peptic ulcer, alcohol and tobacco consumption, stress, the presence of cardiovascular pathology were used as qualitative variables (Table 1). The haplotype blocks were constructed for *MMP-9* gene variants using the «Solid Spine» algorithm (D′ > 0.8) by HaploView program^44^. The logistic regression analyses and adaptive permutation test to adjust for multiple comparisons^45^ were calculated by using the PLINK software^46^. P_perm_ ≤ 0.017 was set to be statistically significant (after the Bonferroni correction based on the numbers of paired comparisons, n = 3: PUD—control, *H. pylori*-positive PUD—control, and *H. pylori*-negative PUD—control).

### Functional SNPs

The polymorphisms associated with PUD and those strongly linked to them (r^2^ ≥ 0.8) were analyzed for their functional significance (non-synonymous SNPs, regulatory potential, eQTLs, and sQTLs)^47^. SNPs in strong linkage disequilibrium (LD) with the PUD-associated variants were identified using HaploReg^40^. Non-synonymous SNPs and their functional predictions were analyzed using the SIFT online tool^48^. The regulatory impact of the candidate *MMP* loci for PUD was evaluated by using HaploReg^40^. The effects of the investigated SNPs on the mRNA levels and splicing QTLs was estimated using the GTEx project data^49^ and the FDR ≤ 0.05 as the significance level. Likewise, eQTL and sQTL values of polymorphisms in strong LD (r^2^ ≥ 0.8) with the PUD-associated loci were estimated^50^.

## Supplementary Information

Supplementary Information 1.

Supplementary Information 2.

Supplementary Information 3.

Supplementary Information 4.

Supplementary Information 5.

Supplementary Information 6.

Supplementary Information 7.

Supplementary Information 8.

Supplementary Information 9.

Supplementary Information 10.

## Data Availability

The data that support the findings of this study are available from the corresponding author upon reasonable request.
