# Supplementary material for: Functionally significant polymorphisms of the MMP-9 gene are associated with peptic ulcer disease in the Caucasian population of Central Russia
Source: Sci Rep. 2021 Jun 29;11:13515. doi: 10.1038/s41598-021-92527-y (PMC8241834; doi:10.1038/s41598-021-92527-y)
Supplement: Supplementary file 1 — Supplementary Information 1. [file 41598_2021_92527_MOESM1_ESM.doc]

**References**

1. Bradbury PA, Zhai R, Hopkins J, et al. Matrix metalloproteinase 1, 3 and 12 polymorphisms and esophageal adenocarcinoma risk and prognosis. *Carcinogenesis*. 2009;30(5):793-798. doi:10.1093/carcin/bgp065
2. Cheung WY, Zhai R, Bradbury P, et al. Single nucleotide polymorphisms in the matrix metalloproteinase gene family and the frequency and duration of gastroesophageal reflux disease influence the risk of esophageal adenocarcinoma. *Int J Cancer*. 2012;131(11):2478-2486. doi:10.1002/ijc.27541
3. Devulapalli K, Bhayal AC, Porike SK. Role of interstitial collagenase gene promoter polymorphism in the etiology of gastric cancer. Saudi J Gastroenterol. 2014; 20(5):309–314. doi:10.4103/1319-3767.141693.
4. Guan X, Wang X, Luo H, Wu J, Zhang X, Wu J. Matrix metalloproteinase 1, 3, and 9 polymorphisms and esophageal squamous cell carcinoma risk. Med Sci Monit. 2014 Nov 13;20:2269-74. doi: 10.12659/MSM.892413.
5. Hellmig S, Ott S, Rosenstiel P, Robert Fölsch U, Hampe J, Schreiber S. Genetic variants in matrix metalloproteinase genes are associated with development of gastric ulcer in H. Pylori infection. Am J Gastroenterol. 2006;101:29–35.
6. Hu C, Weng F, Li L, Dai W, Yan J, Peng L, Zhou R. Association between MMP-9 -1562 C/T polymorphism and susceptibility to digestive cancers: A meta-analysis. Gene. 2018 Oct 5;673:88-94. doi: 10.1016/j.gene.2018.06.025.
7. Kim JH, Pyun JA, Lee KJ, Cho SW, Kwack KB. [Study on association between single nucleotide polymorphisms of MMP7, MMP8, MMP9 genes and development of gastric cancer and lymph node metastasis]. Korean J Gastroenterol. 2011 Nov 25;58(5):245-51. Korean. doi: 10.4166/kjg.2011.58.5.245.
8. Li X, Qu L, Zhong Y, Zhao Y, Chen H, Daru L. Association between promoters polymorphisms of matrix metalloproteinases and risk of digestive cancers: a meta-analysis. *J Cancer Res Clin Oncol*. 2013;139(9):1433-1447. doi:10.1007/s00432-013-1446-9
9. Lin Y, Liu J, Jin L, Jiang Y. Polymorphisms in matrix metalloproteinases 2, 3, and 8 increase recurrence and mortality risk by regulating enzyme activity in gastric adenocarcinoma. *Oncotarget*. 2017;8(62):105971-105983. Published 2017 Nov 20. doi:10.18632/oncotarget.22516
10. Matsumura S, Oue N, Nakayama H, et al. A single nucleotide polymorphism in the MMP-9 promoter affects tumor progression and invasive phenotype of gastric cancer. *J Cancer Res Clin Oncol*. 2005;131(1):19-25. doi:10.1007/s00432-004-0621-4
11. Montazeri Z, Li X, Nyiraneza C, et al. Systematic meta-analyses, field synopsis and global assessment of the evidence of genetic association studies in colorectal cancer. *Gut*. 2020;69(8):1460-1471. doi:10.1136/gutjnl-2019-319313
12. Okada R, Naito M, Hattori Y, Seiki T, Wakai K, Nanri H, Watanabe M, Suzuki S, Kairupan TS, Takashima N, Mikami H, Ohnaka K, Watanabe Y, Katsuura-Kamano S, Kubo M, Hamajima N, Tanaka H Matrix metalloproteinase 9 gene polymorphisms are associated with a multiple family history of gastric cancer. Gastric Cancer. 2017;20(2):246-253. doi: 10.1007/s10120-016-0608-2.
13. Peng B, Cao L, Ma X, Wang W, Wang D, Yu L. Meta-analysis of association between matrix metalloproteinases 2, 7 and 9 promoter polymorphisms and cancer risk. *Mutagenesis*. 2010;25(4):371-379. doi:10.1093/mutage/geq015
14. Peng Q, Xu Y. Association between promoter polymorphisms of matrix metalloproteinase-1 and risk of gastric cancer. Onco Targets Ther. 2015;8:2519–2526. doi:10.2147/OTT.S83004.
15. Peng Z, Jia J, Gong W, et al. The association of matrix metalloproteinase-9 promoter polymorphisms with gastric cancer risk: a meta-analysis. *Oncotarget*. 2017;8(58):99024-99032. doi:10.18632/oncotarget.20931
16. Shaimardanova E.Kh.,. Nurgalieva A.Kh., Khidiyatova I.M., Gabbasova L.V., Kuramshina O.A., Kryukova A.Ya., Sagitov R.B., Munasypov F.R., Khusnutdinova E.K. The role of allelic genes of matrix metalloproteinases and their tissue inhibitors in the development of peptic ulcer. Genetics. 2016;52(3): 364 doi: 10.7868 / S0016675816020119
17. Shan QW, Jing CX, Wang LL, Lu ZL, Tang Q, Yun X, Lian SJ. Relationship between gene polymorphisms in MMP-9 and Helicobacter pylori-related upper gastrointestinal disease in children. Zhongguo Dang Dai Er Ke Za Zhi. 2010 Apr;12(4):262-6. Chinese.
18. Tian J, Liu G, Zuo C, Liu C, He W, Chen H. Genetic polymorphisms and gastric cancer risk: a comprehensive review synopsis from meta-analysis and genome-wide association studies. *Cancer Biol Med*. 2019a;16(2):361-389. doi:10.20892/j.issn.2095-3941.2018.0290
19. Tian J, Liu C, Liu G, Zuo C, Chen H. Cumulative evidence for association between genetic polymorphisms and esophageal cancer susceptibility: A review with evidence from meta-analysis and genome-wide association studies. *Cancer Med*. 2019b;8(3):1289-1305. doi:10.1002/cam4.1972
20. Wu J, Zhang L, Luo H, Zhu Z, Zhang C, Hou Y. Association of matrix metalloproteinases-9 gene polymorphisms with genetic susceptibility to esophageal squamous cell carcinoma. DNA Cell Biol. 2008 Oct;27(10):553-7. doi: 10.1089/dna.2008.0732.
21. Yang MD, Lin KC, Lu MC, et al. Contribution of matrix metalloproteinases-1 genotypes to gastric cancer susceptibility in Taiwan. *Biomedicine (Taipei)*. 2017;7(2):10. doi:10.1051/bmdcn/2017070203
22. Yeh YC, Cheng HC, Chang WL, Yang HB, Sheu BS. Matrix metalloproteinase-3 promoter polymorphisms but not dupA-H. pylori correlate to duodenal ulcers in H. pylori-infected females. BMC Microbiol. 2010 Aug 13;10:218. doi: 10.1186/1471-2180-10-218.
23. Zhang LY, Ren KW. Meta-analysis of MMP2 -1306T allele as a protective factor in digestive cancer. Arch Med Res. 2011 Apr;42(3):239-43. doi: 10.1016/j.arcmed.2011.04.013.
24. Zhang L, Xi RX, Zhang XZ.Matrix metalloproteinase variants associated with risk and clinical outcome of esophageal cancer. Genet Mol Res. 2015;14(2):4616-24. doi: 10.4238/2015.May.4.20.
