# Supplementary material for: Functionally significant polymorphisms of the MMP-9 gene are associated with peptic ulcer disease in the Caucasian population of Central Russia
Source: Sci Rep. 2021 Jun 29;11:13515. doi: 10.1038/s41598-021-92527-y (PMC8241834; doi:10.1038/s41598-021-92527-y)
Supplement: Supplementary file 2 — Supplementary Information 2. [file 41598_2021_92527_MOESM2_ESM.docx]

Supplementary table 1

The allele and genotype frequencies of the studied SNPs in the PUD and control groups

| Chr | SNP | Gene | Minor allele | Major allele | Minor allele frequency | Number of the studied chromosomes | Genotype distribution* | H_o_ | H_e_ | Р_HWE_ |
| --- | --- | --- | --- | --- | --- | --- | --- | --- | --- | --- |
| PUD patients (n=798) | | | | | | | | | | |
| 11 | rs1940475 | *MMP-8* | T | C | 0.484 | 1580 | 204/357/229 | 0.452 | 0.499 | 0.069 |
| 11 | rs1799750 | *MMP-1* | 2G | 1G | 0.451 | 1536 | 166/361/241 | 0.470 | 0.495 | 0.303 |
| 11 | rs679620 | *MMP-3* | T | C | 0.494 | 1576 | 199/381/208 | 0.484 | 0.450 | 0.546 |
| 16 | rs243865 | *MMP-2* | T | C | 0.249 | 1556 | 56/276/446 | 0.354 | 0.374 | 0.342 |
| 20 | rs3918242 | *MMP-9* | T | C | 0.165 | 1568 | 14/231/539 | 0.295 | 0.276 | 0.267 |
| 20 | rs3918249 | *MMP-9* | C | T | 0.399 | 1560 | 111/401/268 | 0.514 | 0.480 | 0.205 |
| 20 | rs17576 | *MMP-9* | G | A | 0.413 | 1588 | 145/365/284 | 0.460 | 0.485 | 0.300 |
| 20 | rs3787268 | *MMP-9* | A | G | 0.222 | 1576 | 33/284/471 | 0.360 | 0.345 | 0.382 |
| 20 | rs2250889 | *MMP-9* | G | C | 0.098 | 1572 | 12/130/644 | 0.165 | 0.176 | 0.243 |
| 20 | rs17577 | *MMP-9* | A | G | 0.169 | 1544 | 14/233/525 | 0.302 | 0.281 | 0.202 |
| Control group (n=347) | | | | | | | | | | |
| 11 | rs1940475 | *MMP-8* | T | C | 0.494 | 692 | 92/158/96 | 0.456 | 0.499 | 0.107 |
| 11 | rs1799750 | *MMP-1* | 2G | 1G | 0.479 | 678 | 85/155/99 | 0.457 | 0.499 | 0.127 |
| 11 | rs679620 | *MMP-3* | T | C | 0.504 | 690 | 89/170/86 | 0.492 | 0.500 | 0.829 |
| 16 | rs243865 | *MMP-2* | T | C | 0.249 | 686 | 24/123/196 | 0.358 | 0.374 | 0.470 |
| 20 | rs3918242 | *MMP-9* | T | C | 0.169 | 686 | 11/94/238 | 0.274 | 0.281 | 0.699 |
| 20 | rs3918249 | *MMP-9* | C | T | 0.377 | 690 | 57/146/142 | 0.423 | 0.469 | 0.067 |
| 20 | rs17576 | *MMP-9* | G | A | 0.361 | 692 | 46/158/142 | 0.456 | 0.461 | 0.907 |
| 20 | rs3787268 | *MMP-9* | A | G | 0.207 | 690 | 14/115/216 | 0.333 | 0.328 | 0.870 |
| 20 | rs2250889 | *MMP-9* | G | C | 0.123 | 684 | 9/66/267 | 0.193 | 0.215 | 0.072 |
| 20 | rs17577 | *MMP-9* | A | G | 0.172 | 680 | 13/91/236 | 0.267 | 0.284 | 0.255 |

Supplementary table 1 (continued)

The allele and genotype frequencies of the studied SNPs in the PUD and control groups

| Chr | SNP | Gene | Minor allele | Major allele | Minor allele frequency | Number of the studied chromosomes | Genotype distribution* | H_o_ | H_e_ | Р_HWE_ |
| --- | --- | --- | --- | --- | --- | --- | --- | --- | --- | --- |
| *H. pylori*-positive PUD patients (n=404) | | | | | | | | | | |
| 11 | rs1940475 | *MMP-8* | T | C | 0.481 | 796 | 102/179/117 | 0.450 | 0.499 | 0.201 |
| 11 | rs1799750 | *MMP-1* | 2G | 1G | 0.448 | 772 | 84/178/124 | 0.461 | 0.494 | 0.382 |
| 11 | rs679620 | *MMP-3* | T | C | 0.482 | 796 | 96/192/110 | 0.482 | 0.499 | 0.670 |
| 16 | rs243865 | *MMP-2* | T | C | 0.250 | 784 | 32/132/228 | 0.336 | 0.375 | 0.180 |
| 20 | rs3918242 | *MMP-9* | T | C | 0.191 | 792 | 4/143/249 | 0.361 | 0.309 | 0.019 |
| 20 | rs3918249 | *MMP-9* | C | T | 0.437 | 784 | 65/213/114 | 0.543 | 0.492 | 0.147 |
| 20 | rs17576 | *MMP-9* | G | A | 0.458 | 800 | 90/186/124 | 0.465 | 0.496 | 0.393 |
| 20 | rs3787268 | *MMP-9* | A | G | 0.239 | 800 | 23/145/232 | 0.363 | 0.363 | 1.000 |
| 20 | rs2250889 | *MMP-9* | G | C | 0.094 | 788 | 4/66/324 | 0.167 | 0.170 | 0.680 |
| 20 | rs17577 | *MMP-9* | A | G | 0.199 | 776 | 6/142/240 | 0.366 | 0.318 | 0.042 |
| *H. pylori*-negative PUD patients (n=394) | | | | | | | | | | |
| 11 | rs1940475 | *MMP-8* | T | C | 0.487 | 784 | 102/178/112 | 0.454 | 0.499 | 0.200 |
| 11 | rs1799750 | *MMP-1* | 2G | 1G | 0.452 | 764 | 82/181/119 | 0.474 | 0.495 | 0.661 |
| 11 | rs679620 | *MMP-3* | T | C | 0.471 | 780 | 89/189/112 | 0.485 | 0.498 | 0.774 |
| 16 | rs243865 | *MMP-2* | T | C | 0.249 | 772 | 24/144/218 | 0.373 | 0.373 | 1.000 |
| 20 | rs3918242 | *MMP-9* | T | C | 0.139 | 776 | 10/88/290 | 0.227 | 0.239 | 0.383 |
| 20 | rs3918249 | *MMP-9* | C | T | 0.363 | 776 | 48/186/154 | 0.479 | 0.463 | 0.756 |
| 20 | rs17576 | *MMP-9* | G | A | 0.367 | 788 | 55/179/160 | 0.454 | 0.465 | 0.759 |
| 20 | rs3787268 | *MMP-9* | A | G | 0.206 | 776 | 10/140/238 | 0.361 | 0.327 | 0.192 |
| 20 | rs2250889 | *MMP-9* | G | C | 0.102 | 784 | 8/64/320 | 0.163 | 0.183 | 0.121 |
| 20 | rs17577 | *MMP-9* | A | G | 0.138 | 768 | 8/90/286 | 0.234 | 0.238 | 0.764 |

Note: * minor allele homozygotes / heterozygotes / major allele homozygotes
